# Impaired Spatial Learning and Memory in Middle-Aged Mice with Kindling-Induced Spontaneous Recurrent Seizures

**DOI:** 10.3389/fphar.2019.01077

**Published:** 2019-09-24

**Authors:** Haiyu Liu, Kurt R. Stover, Nila Sivanenthiran, Jonathan Chow, Chloe Cheng, Yapeng Liu, Stellar Lim, Chiping Wu, Donald F. Weaver, James H. Eubanks, Hongmei Song, Liang Zhang

**Affiliations:** ^1^Department of Neurosurgery, The First Hospital of Jilin University, Jilin, China; ^2^Krembil Research Institute, University Health Network, Toronto, ON, Canada; ^3^Department of Chemistry, University of Toronto, Toronto, ON, Canada; ^4^Department of Medicine, University of Toronto, Toronto, ON, Canada; ^5^Department of Physiology, University of Toronto, Toronto, ON, Canada; ^6^Department of Surgery, University of Toronto, Toronto, ON, Canada

**Keywords:** epilepsy, hippocampus, kindling, mice, memory

## Abstract

Temporal lobe epilepsy is the most common and often drug-resistant type of epilepsy in the adult and aging populations and has great diversity in etiology, electro-clinical manifestations, and comorbidities. Kindling through repeated brief stimulation of limbic structures is a commonly used model of temporal lobe epilepsy. Particularly, extended kindling can induce spontaneous recurrent seizures in several animal species. However, kindling studies in middle-aged, aging, or aged animals remain scarce, and currently, little is known about kindling-induced behavioral changes in middle-aged/aging animals. We therefore attempted to provide more information in this area using a mouse model of extended hippocampal kindling. We conducted experiments in middle-aged mice (C57BL/6, male, 12–14 months of age) to model new-onset epilepsy in adult/aging populations. Mice experienced twice daily hippocampal stimulations or handling manipulations for 60–70 days and then underwent continuous electroencephalogram (EEG)-video monitoring to detect spontaneous recurrent seizures. Extended kindled mice consistently exhibited spontaneous recurrent seizures with mean incidences of 6–7 events per day, and these seizures featured EEG discharges and corresponding convulsions. The handling control mice showed neither seizure nor aberrant EEG activity. The two groups of mice underwent the Morris water maze test of spatial learning and memory 1–2 weeks after termination of the kindling stimulation or handling manipulation. During visible platform trials, the kindled mice took a longer distance and required more time than the control mice to find the platform. During hidden platform trials, the kindled mice showed no improvement over 5-day trials in finding the platform whereas the control mice improved significantly. During probe tests in which the hidden platform was removed, the kindled mice spent less time than the controls searching in the correct platform location. There were no significant differences between the kindled and control mice with respect to swim speed or total locomotor activity in an open-field test. Together, these observations indicate that the extended kindled mice with spontaneous recurrent seizures are impaired in spatial learning and memory as assessed by the Morris water maze test. We postulate that the extended hippocampal kindling in middle-aged mice may help explore epileptogenic mechanisms and comorbidities potentially relevant to new-onset temporal lobe epilepsy in adult and aging patients. Limitations and confounds of our present experiments are discussed to improve future examinations of epileptic comorbidities in extended kindled mice.

## Introduction

Epilepsy is a disease characterized by an enduring predisposition to generate epileptic seizures and by the neurobiological, cognitive, psychological, and social consequences of this condition (2014 definition, International League Against Epilepsy). Temporal lobe epilepsy is the most common and often drug-resistant type of epilepsy in the adult and aging populations and has great diversity in etiology, electro-clinical properties, and comorbidities (Hauser, 1992; Engel, 1996; Brodie et al., 2009; Ferlazzo et al., 2016). It has been increasingly recognized that the treatment of epilepsy is not restricted to the achievement of seizure freedom but must also include the management of comorbid medical, neurological, psychiatric, and cognitive comorbidities (Kanner, 2016). Kindling through repeated brief stimulation of limbic structures has long been used to model temporal lobe epilepsy and comorbidities (see reviews by Brooks-Kayal et al., 2013; Gorter et al., 2016; Löscher, 2016; Mazarati, 2017; Sutula and Kotloski, 2017). However, kindling studies in middle-aged, aging, or aged animals remain scarce (de Toledo-Morrell et al., 1984; Fanelli and McNamara, 1986; Stover et al., 2017). Currently, little is known about kindling-induced behavioral changes in middle-aged/aging animals.

Classic or extended kindling protocols have been used in previous studies. While classic kindling lasting a few weeks does not induce spontaneous recurrent seizures (SRS), extended kindling is able to induce SRS in monkeys (Wada and Osawa, 1976), dogs (Wauquier et al., 1979), cats (Wada et al., 1974: Gotman 1984; Hiyoshi et al., 1993), rats (Pinel and Rovner, 1978; Pinel, 1983; Milgram et al., 1995; Michalakis et al., 1998; Sayin et al., 2003; Brandt et al., 2004), and mice (Song et al., 2018). SRS induction by extended kindling is generally not associated with gross brain injury, but rather a loss of subgroups of GABAergic interneurons in the hippocampal hilar region (Sayin et al., 2003; but see Brandt et al., 2004). The lack of observed gross brain injury in extended kindled animals is different from poststatus epilepticus models in which SRS emergence is accompanied with pronounced brain damage (Dudek and Staley, 2017; Gorter and van Vliet, 2017; Henshall, 2017; Kelly and Coulter, 2017). As such, the extended kindling model may help explore epileptogenesis and comorbidities in the absence of major brain pathology as is seen in many patients with temporal lobe epilepsy (Ferlazzo et al., 2016).

To date, two published studies are available concerning behavioral changes in extended kindled rats with SRS. One study demonstrated an increase of body weight in amygdala-kindled rats (Löscher et al., 2003) and the other examined performances of amygdala/performant-path-kindled rats in the Morris water maze (MWM) and open-field tests (Cammisuli et al., 1997). Kindled rats in the latter study were impaired during the initial training on hidden platform acquisition but not in retention of platform location in the MWM test. In addition, these animals showed a transient increase in open-field activity. Together, these findings indicate that the extended kindling of the amygdala/performant path in adult rats disrupted the acquisition phase of a spatial memory task and induced a transient elevation in open-field locomotor/exploratory behaviors. The issue remains open as to whether extensive kindling at other limbic structures and in middle-aged/aging animals may lead to more pronounced behavioral deficits.

We explored these issues in the present experiments using a mouse model of extended hippocampal kindling (Song et al., 2018). Specifically, we conducted experiments in middle-aged mice (12–14 months old) to model new-onset epilepsy seen clinically in adult/aging populations. Mice received chronic kindling stimulation or handling manipulations (controls) and then underwent continuous EEG-video monitoring before and following MWM and open-field tests. Our data indicate that middle-aged mice with kindling-induced SRS have pronounced deficits in spatial learning and memory.

## Methods

### Animals

Male C57 black mice (C57BL/6N) were obtained from Charles River Laboratory (Saint-Constant, Quebec, Canada) and housed in a local vivarium. The vivarium was maintained at room temperature between 22°C and 23°C with a 12-h light on/off cycle (lights on at 6:00 am). Mice were housed in group (up to four mice per cage) with food and water *ad libitum*. All experimentations described below were reviewed and approved by the Animal Care Committee of the University Health Network in accordance with the Guidelines of the Canadian Council on Animal Care.

C57 black mice have a maximal lifespan up to 36 months but aging/aged mice often encounter health-related complications including skin lesions, ear infections, and tumors (Flurkey et al., 2007). We therefore chose to start kindling in middle-aged mice (ages 11–12 months) in an attempt to model new-onset epilepsy and comorbidities as seen clinically in adult/aging populations (Brodie et al., 2009; Ferlazzo et al., 2016) while minimizing the health-related complications that are common in aging/aged mice.

### Electrode Construction and Implantation

Electrode construction and implantation were performed as previously described (Jeffrey et al., 2014; Bin et al., 2017). All electrodes were made of polyamide-insulated stainless steel wires (110-μm outer diameter; Plastics One, Roanoke, Virginia, USA). Twisted bipolar electrodes were used for stimulation and/or recording. Surgeries were performed under isoflurane anesthesia. A stereotaxic frame and micromanipulators were used for electrode placement. Implanted electrodes were secured onto the skull using the glue-based method. Each mouse was implanted with two pairs of bipolar electrodes; one positioned to the hippocampal CA3 (bregma −2.5 mm, lateral 3.0 mm, and depth 3.0 mm; Franklin and Paxinos, 1997) for kindling stimulation and local recordings and other targeted to the ipsilateral/contralateral parietal cortex (bregma −0.5 mm, lateral 2.0 mm, and depth 0.5 mm). A reference electrode was positioned to a frontal area (bregma +1.5 mm, lateral 1.0 mm, and depth 0.5 mm). The locations of implanted electrodes were verified by later histological assessments when suitable.

### Hippocampal Kindling

A train of stimuli at 60 Hz for 2 s was used for hippocampal kindling stimulation (Reddy and Rogawski, 2010; Jeffrey et al., 2014; Bin et al., 2017; Stover et al., 2017; Song et al., 2018). Constant current pulses with monophasic square waveforms, a pulse duration of 0.5 ms, and current intensities of 10–150 μA were generated by a Grass stimulator and delivered through a photoelectric isolation unit (model S88, Grass Medical Instruments, Warwick, Rhode Island, USA). An ascending series was used to determine the threshold of evoked afterdischarges in individual mice. In the assessing series, a stimulation train with incremental current intensities (10 μA per step) was applied every 30 min. The lowest current that elicited an afterdischarge event of ≥5 s was considered the afterdischarge threshold. Stimulations on subsequent days used a stimulation current intensity at 25% above the threshold value (Reddy and Rogawski, 2010). Our goal was to keep constant stimulation intensity throughout the extended kindling period. However, the initial stimulation intensity often became inconsistent in evoking afterdischarges after ≥45 days of kindling experiments, which might be a result of contamination of the implanted electrodes. Due to this this complication, stronger stimulation intensities (40–80 μA above the initial intensities) were used in subsequent experiments.

Kindling stimuli were applied twice daily between 10 AM and 5 PM and ≥5 h apart (Pinel and Rovner, 1978; Pinel, 1983; Milgram et al., 1995; Michalakis et al., 1998; Sayin et al., 2003; Brandt et al., 2004). Each stimulation episode lasted for several minutes during which the mouse was placed in a glass container for EEG-video monitoring (Stover et al., 2017; Song et al., 2018). Control mice were handled twice daily for 60 consecutive days.

### Continuous EEG-Video Monitoring

Concurrent EEG recordings and video monitoring were done as previously described (Bin et al., 2017). Briefly, an implanted mouse was placed in a modified cage with food and water *ad libitum*. A slip-ring commutator was mounted atop the cage and connected to the mouse *via* flexible cables. A webcam was placed near the cage to capture mouse’s motor seizures. Dim lighting was used for webcam monitoring during the lights-off period. EEG and video data were collected roughly 24 h daily and for 2–3 consecutive days per session. A cursor auto-click program (Mini Mouse Macro program; http://www.turnssoft.com/mini-mouse-macro.html) was used to save data every 2 h.

Baseline EEG-video monitoring of 24 h was performed ≥1 week after electrode implantation. Similar monitoring was made after the 80^th^, 100^th^, 120^th^, and/or 140^th^ stimulation to assess SRS commencement. No further kindling stimulation was applied if ≥2 SRS events/day were detected, and additional monitoring for 24–48 h was performed to assess initial SRS daily incidences. Continuous EEG-video monitoring for 24–72 h was performed after behavioral tests to determine whether SRS persisted in individual mice. The control mice were monitored for 24 h after 60 days of handling manipulations.

Differential recordings through twisted bipolar electrodes were used to sample local EEG signals (Jeffrey et al., 2014; Bin et al., 2017; Stover et al., 2017; Song et al., 2018). Mono-polar EEG recordings were used only if the differential recordings were unsuccessful. Signals were collected using two-channel or one-channel microelectrode AC amplifiers with extended head-stages (model 1800 or 3000, AM Systems; Sequim, Washington, USA). Evoked afterdischarges of the stimulated hippocampal CA3 were captured using the model 3000 amplifier *via* TTL-gated switches between recording and stimulating modes. These amplifiers were set with an input frequency band of 0.1–1,000 Hz and amplification gain of 1,000. Amplifier output signals were digitized at 5,000 Hz (Digidata 1440A or 1550, Molecular Devices; Sunnyvale, California, USA). Data acquisition, storage, and analyses were done using pCLAMP software (Version 10; Molecular Devices).

### Measurements of Discharges, Interictal Spikes and Motor Seizures

Evoked afterdischarges and spontaneous ictal discharges were recognized by repetitive spike waveforms with amplitudes approximately two times of background signals and durations of ≥5 s (Jeffrey et al., 2014; Bin et al., 2017; Stover et al., 2017; Song et al., 2018). Interictal spikes were recognized by large amplitudes (≥8 times of standard deviation of background signals) and simple/complex waveforms and durations of 30–250 ms. Spikes were measured from 30-min EEG segments for individual mice. These segments were collected at ≥2 h after an ictal discharge to minimize post-ictal influences on interictal activities. The event detection function (threshold search method) of pCLAMP software was used to automatically detect spikes, and detected events were then visually inspected, and false events were rejected (El-Hayek et al., 2013; Song et al., 2018). EEG data analyses were made independently by three researchers (HYL, HMS, and LZ). Consensus on disputed events was reached after discussion.

Evoked and spontaneous motor seizures were scored using the Racine scale modified for mice (Racine, 1972; Reddy and Rogawski, 2010). Briefly, stage 0—no response or behavioral arrest, stage 1—chewing or facial movement, stage 2—chewing and head nodding, stage 3—unilateral or bilateral forelimb clonus, stage 4—bilateral forelimb clonus and rearing, and stage 5—rearing and falling with limb clonus. Evoked and spontaneous motor seizures were assessed independently by several researchers (JC, NS, CC, YPL, and SL). The concordance rates for recognizing stage 4–5 seizures were ≥90% among these researchers.

### Morris Water Maze (MWM) Test

A dark blue pool with 120 cm in diameter, and 50 cm in depth was used. The pool was placed in a quiet room and filled with white-colored water. The water was equilibrated to room temperature (between 22°C and 23°C) for at least 48 h before the MWM test. Colored papers with a variety of different shapes were posted around the pool as visual cues. A platform of 10 cm in diameter was used. For hidden platform trials, the platform was positioned 1.5 cm below water surface. A Panlab tracking system (Harvard Apparatus, Quebec, Canada) was used to monitor animal’s behaviors in the pool. The MWM test was performed in the period of 11AM–3PM to minimize circadian effects. The extended kindled and control mice were tested in same days, and testing sequences for individual mice were altered in each test day.

The MWM test was performed 1–2 weeks after ending the kindling stimulation or handling manipulation. A protocol with 3 days of visible platform trials, 5 days of hidden platform trials, and two probe tests on hidden days 3 and 5 were employed (Vorhees and Williams, 2006; Stover and Brown, 2012; **Figure 2A**). In the visible and hidden platform trials, individual mice underwent four trials per day, and the maximal time for each trial was 90 s. If mice did not find the platform within 90 s, they were guided to the platform by the experimenter’s hand and allowed to stay on the platform for 15 s. For the probe tests in which the platform was removed from the pool, individual mice underwent a trial of 60 s. If mice exhibited convulsions shortly before or during a trial, they were allowed to recover for 20–30 min before next trial. Any trial interfered with convulsions were excluded from analysis. Before the third day of the hidden phase and the day following the last hidden trials, the mice were subjected to a probe trial where the platform was removed, and the mice were allowed to swim in the pool for a single 60-second trial. Distances and latencies to find the platform, swim speed during the visible and hidden platform trials, and the time in searching the correct quadrant during the probe trials were analyzed. Group data for the extended kindled and control mice were compared.

### Open-Field Test

The open-field test of 1-h duration was conducted 4–7 days after the MWN test and in the period of 10AM–1PM to minimize circadian effects. Each mouse was examined only once to prevent acclimation to the open-field apparatus. Care was taken to clean the plexiglass arena with 75% alcohol and water before each test to avoid odor interference from preceding test.

We used a plexiglass open-field arena (20×30 cm; Jugloff et al., 2008; Wither et al., 2013). The arena is surrounded by a housing frame that accommodates an array of 24 infrared beams forming a grid across two levels. The lower grid measures X–Y movement while the upper grid measures rearing movement. As the mouse moves, a beam is broken and registered as an activity count, depending on the timing and number of concurrent beams broken; different types of activity are recorded. Several behavioral parameters are assessed using this system. Total static or mobile count means total beam breaks in which changes in mouse’s position are below or above the mobile threshold. Total rearing or central rearing count denotes the upper beam breaks detected in entire arena or in arena center. Total activity count represents total beam breaks by static, mobile, and rearing behaviors. With respect to static, mobile, or rearing time, the measure is incremented every time as the mouse is engaged in a type of activity in any given second. Active time means the total activity time including static, mobile, and rearing.

### Statistical Analyses

Statistical tests were conducted using Prism 6 (GraphPad Software, San Diego, California, USA) or SigmaPlot software (Systat Software Inc., San Jose, California, USA). Student’s t-test or Mann–Whitney rank-sum test was used for two-group comparisons. A one-way ANOVA or one-way ANOVA on ranks was used for multiple group comparisons, followed by a multiple comparison Holm–Sidak or Tukey test. A mixed repeated ANOVA was used for within group comparisons. Pearson or Spearman rank-order test was used for correlation analysis. Data are presented as means and standard error of the mean (SEM) throughout the text and figures. Statistical significance was set at p < 0.05.

## Results

### General Behaviors and SRS Assessments

We collected data from 12 kindled mice and 12 handling control mice. The hippocampal kindling or handling manipulation over 9–10 weeks did not cause evident disruption in ambient cage behaviors or significant changes in body weights. Measured body weights were 29.1 ± 1.1 and 28.0 ± 0.8 g (p = 0.408) before kindling and after ending the kindling stimulation and 30.9 ± 1.4 and 29.4 ± 1.7 g (p = 0.487) before handling and after ending the handling manipulation. There is a discrepancy between our present observations and the previous study demonstrating excessive body weight gain in rats following extended amygdala kindling (Löscher et al., 2003). This may be due largely to a difference in kindling sites as the amygdala is known to play an important role in regulating food intake although the hippocampus may complement amygdala functions (Coppin, 2016).

**Figure 1:**
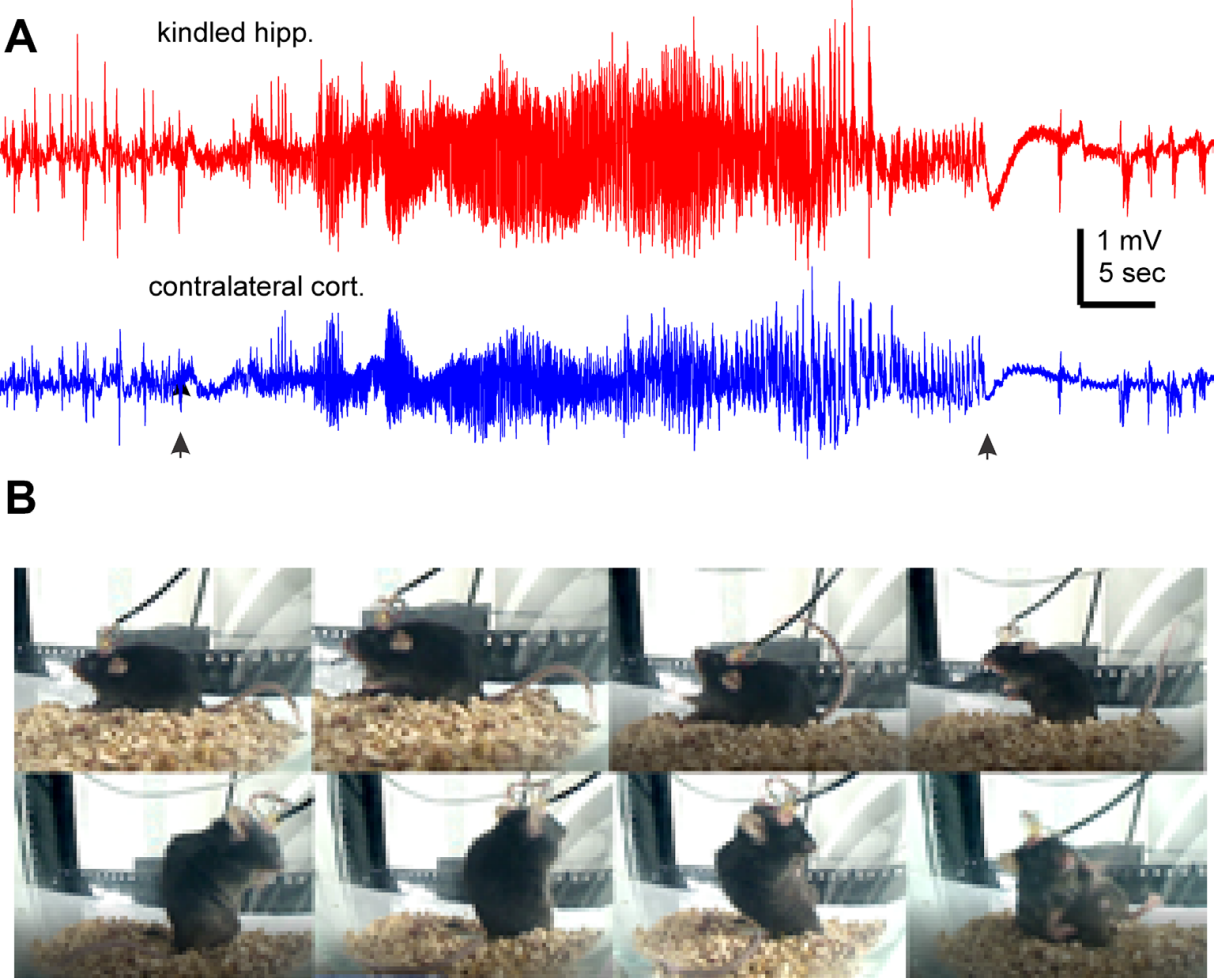
Representative EEG discharges and corresponding motor seizure. EEG traces and images were collected from a mouse the first day after termination of kindling stimulation. **(A)** Ictal discharges were recorded from of the kindled hippocampus and contralateral parietal cortex. Filled arrows denote the onset and tarnation of discharges. **(B)** Sequential images (from top-left to bottom right) show a stage-5 seizure.

We used a kindling protocol with twice daily stimulations in the present experiments. Mice were fully kindled following 19 ± 1.7 hippocampal stimulations (ranging 9 to 27 stimuli, n = 12) as indicated by three consecutively evoked stage-5 motor seizures (Jeffrey et al., 2014; Stover et al., 2017; Song et al., 2018). These mice exhibited SRS following 107.8 ± 5.1 hippocampal stimulations (ranging from 85 to 140 stimuli). Evoked hippocampal afterdischarges cumulative to SRS were 3,779.2 ± 399.0 s (ranging from 2,712 to 6,882 s), and cumulative Racine scales for corresponding motor seizures were 551.2 ± 56.8 (ranging from 390 to 948). Detected *via* continuous EEG-video monitoring in the first 2–3 days after ending the kindling stimulation, SRS incidences were 7.1 ± 0.7 events/day (ranging from 3.5 to 11 daily events), corresponding hippocampal ictal discharges were 45.6 ± 1.4 s in duration, and motor seizure scores were 3.54 ± 0.06 on the modified Racine scale. There was no significant correlation between the stimulation numbers needed to reach the fully kindled or SRS state and the initial SRS incidence (p > 0.05). The cumulative measures of evoked after discharges or motor seizures were also uncorrelated with the initial SRS incidences (p > 0.05). **Figures 1A, B** illustrates representative EEG discharges and video-captured images collected from a kindled mouse in the first day after termination of kindling stimulation.

In addition to the ictal discharges, the kindled mice exhibited repetitive interictal spikes that manifested during immobility or sleep but were also detectable in movement or exploratory behaviors (Song et al., 2018). We measured hippocampal interictal spikes as they were more frequent and robust than cortical spikes. Spike analyses were made in 30-min data segments that were collected during immobility/sleep and in the first 2–3 days after ending the kindling stimulation. The mean inter-spike intervals were in a range of 2.1 ± 0.053 to 12.9 ± 1.7 s (12 mice).

The kindled mice exhibit 6.6 ± 0.6 SRS events/day while being monitored 1–2 weeks after the behavioral tests. There was no significant difference between the initial and later SRS incidence measures (p = 0.672). Hippocampal interictal spikes were observed in all the kindled mice following the behavioral tests, but spike amplitudes and background signals were decreased. The latter might have largely resulted from contamination in implanted electrodes which complicated spike detection. Hippocampal interictal spikes were reliably measured from four kindled mice before and following the behavioral tests, showing no significant difference in inter-spike intervals (5.8 ± 2.4 and 4.3 ± 0.8 s, n = 4, p = 0.568). Together, these observations suggest that SRS and interictal spikes might persist in extended kindled mice.

In contrast to the extended kindled mice, the control mice showed neither seizure nor aberrant EEG activity while being examined *via* 24-h EEG-video monitoring after ending the handling manipulation.

### Morris Water Maze (MWM) Test

**Figure 2:**
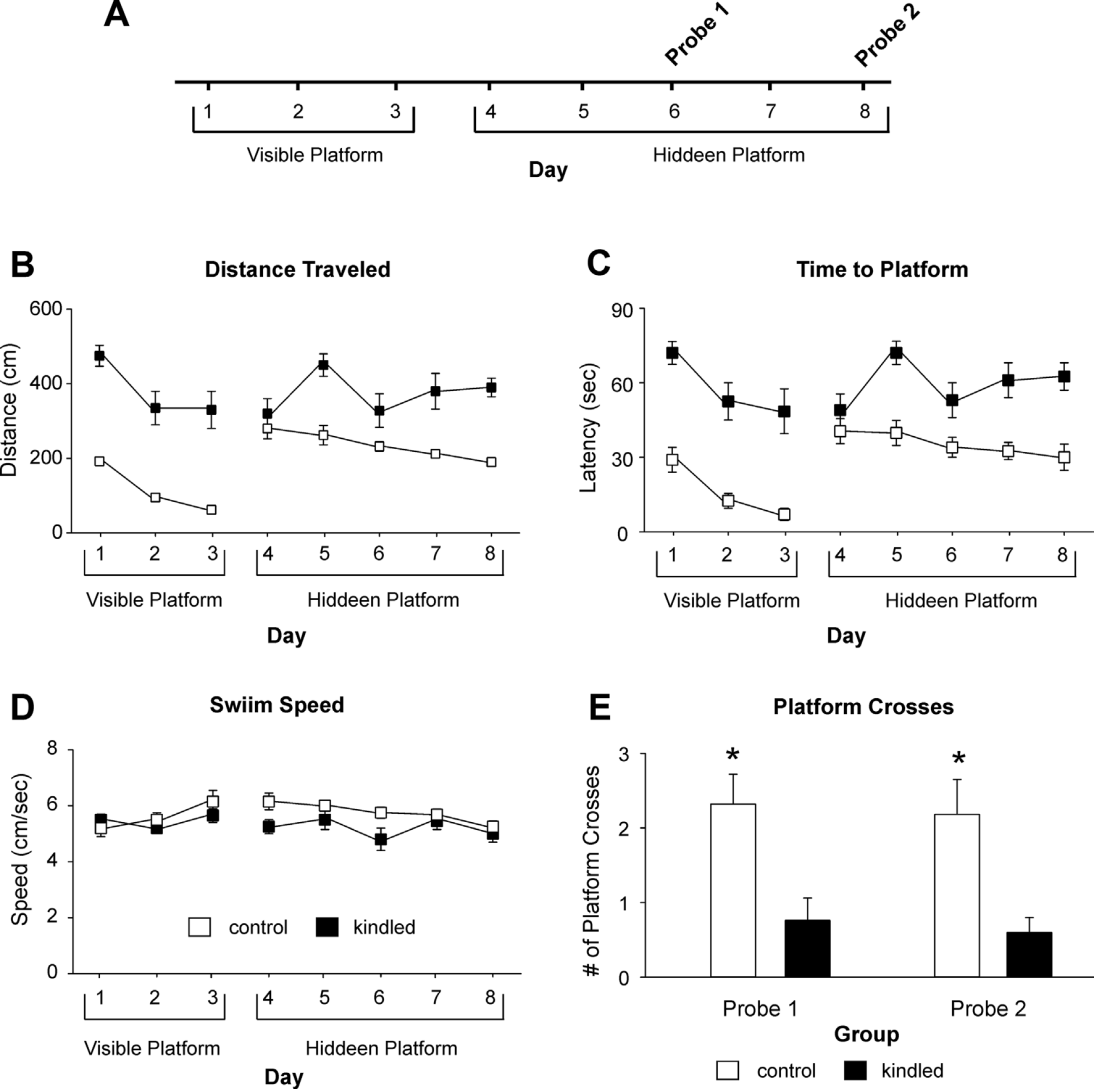
Distances and latency Measures in the MWM test. **(A)** A schematic layout of the MWM protocol. The visible and hidden platform trials consisted of four trials per day and maximal 90 s per trial. The probe tests were one trial per day and 60 s per trial with the platform removed. Data in **(B**, **E)** (means ± SEM) were collected from 12 extended kindled mice and 12 handling control mice. **(B**, **C)** Distances and latencies needed to reach the platform. There were significant group differences in the distance and latency measures during the visible and hidden platform trials. **(D)** Swim speeds measured during the visible and hidden platform trials. There were no significant group differences in these measures. **(E)** Crosses of the platform location during the first and second probe test. The kindled mice crossed the platform location significantly less frequently (denoted by *) than the control mice.

The MWM test was conducted 1–2 weeks after the initial EEG-video monitoring. We used a protocol with 3 days of visible platform trials, 5 days of hidden platform trials, and 2 probe tests on days 3 and 5 of the hidden platform trials (**Figure 2A**). During the visible platform trials, both the kindled and control mice were significantly improved in performance over the 3-day trials, as there were day/trial-dependent reductions in swim distance [F(2,44) = 16.089, p < 0.001] and time [F(2,44) = 15.055, p < 0.001] in finding the platform (**Figures 2B, C**). However, the kindled mice swam significantly longer distances [F(1,22) = 28.161, p < 0.001] and took significantly longer times [F(1,22) = 30.671, p < 0.001] relative to the controls to find the platform.

During hidden platform trials, the kindled mice also swam significantly longer distances and spent more time than the control mice to reach the platform [F(1,22) = 17.280 and 17.086; p < 0.001]. Over the 5-day trials, the kindled mice showed no performance improvement as there were no day/trial-dependent reductions in swim distance and time [F(4,88) = 1.888 and 8.208, p = 0.120 and 0.074], whereas the control mice improved moderately but significantly over the 5-day trials [F(4,88) = 2.770, p = 0.032; **Figures 2B, C**].

In both the visible and hidden platform trials, there was no significant difference between the kindled and control mice with respect to swim speed [F(1,22) <1 and 2.034, p = 0.973 and 0.168] nor speed changes over the 3-day or 5-day trials [F(4,88) = 1.994 and <1, p = 0.102 and 0.543; **Figure 2D**].

**Figure 3:**
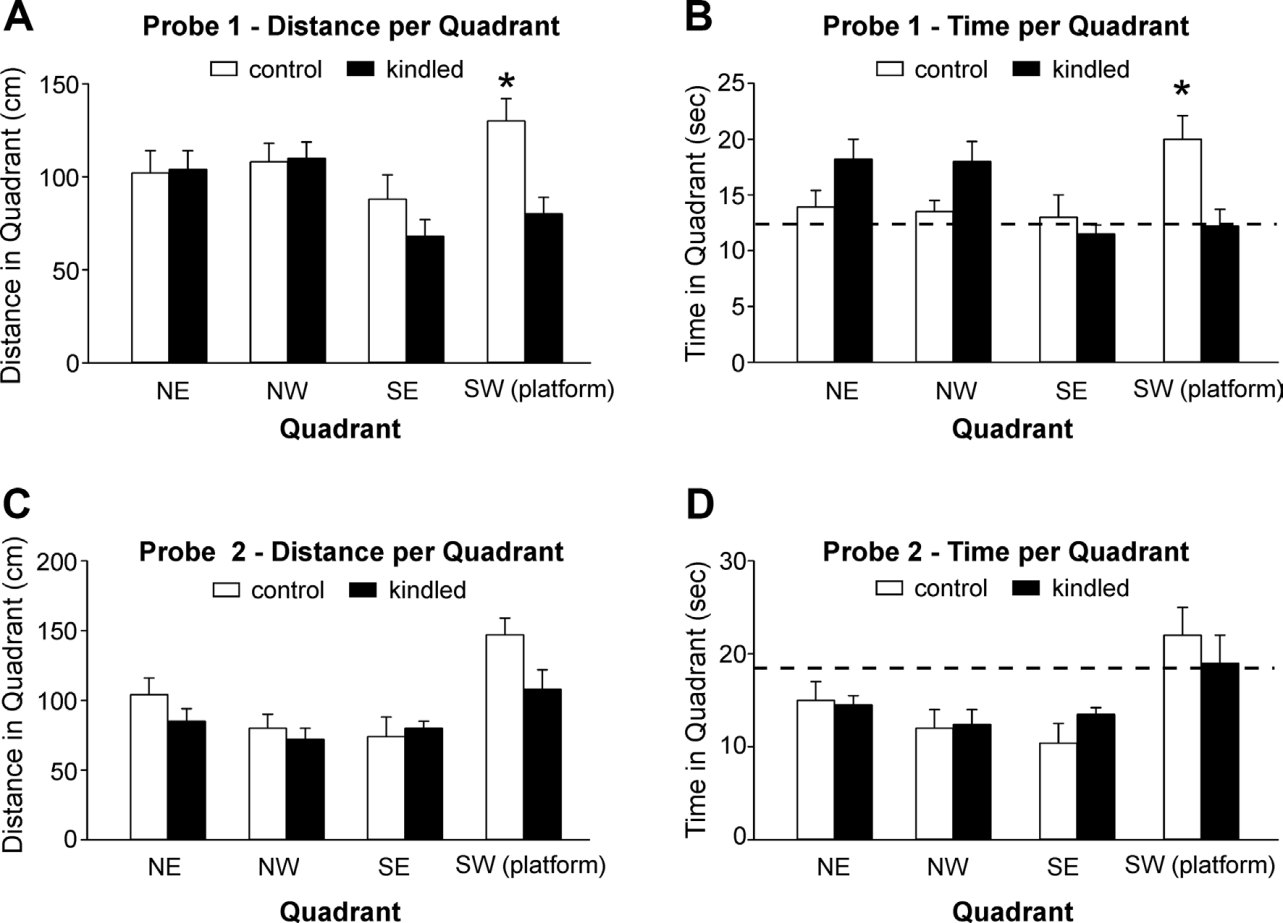
Measures for probe tests. Data (means ± SEM) were collected from the extended kindled and handling control mice (n = 12 each group). **(A**, **B)** Distances traveled and time spent in each of the quadrants during the first probe trial. **(C**, **D)** The same measures collected during the second probe trial. Dotted lines represent chance performance. Note that the kindled mice traveled shorter distances and spent less time in the correct quadrant as compared to the controls. Significant differences between the control and extended kindled mice were denoted by *.

During the probe tests 1 and 2 where the platform was removed from the south-west quadrant, the extended kindled mice crossed the south-west quadrant significantly less frequently than the control mice [F(1,22) = 10.704 and 9.210, p = 0.003 and 0.006] (**Figures 2D, E**). For swim distance per quadrant in the first probe test, there was a significant effect of kindling group [F(1,22) = 7.505, p = 0.012], quadrant [F(3,66) = 3.151, p = 0.031], and a kindling group by quadrant interaction [F(3,66) = 3.418, p = 0.022], as the control mice travelled a greater distance in the correct quadrant than then control mice (**Figure 3A**). There was no effect of kindling group [F(1,22) <1] or quadrant [F(3,66) = 1.710, p = 0.174] in time spent in each quadrant during the first probe trial, but there was a kindling group by quadrant interaction [F(3,66) = 3.815, p = 0.014] as the kindled mice spent less time in the correct quadrant than the control mice while there was no difference in time spent in other quadrants (**Figure 3B**). This indicated that the kindled mice did not remember the platform location during the first probe test.

For both swim distance [F(3,63) = 9.011, p < 0.001] and time [F(3,63) = 6.479, p < 0.001] per quadrant in the second probe test, there was only an effect of quadrant, as all mice favored the correct quadrant, and there were no significant differences between kindling groups or interactions (**Figures 3C, D**). This indicated that all mice remembered the platform location equally well.

### Open-Field Test

The open-field test was conducted 4–7 days after the MWM test. We used a protocol with 1-h duration (Jugloff et al., 2008; Wither et al., 2013) and obtained multiple measures from the open-field test (**Table 1**). Data were collected from 9 of the 12 kindled mice and from 10 of the 12 control mice because 3 kindled mice encountered convulsive seizures during the open-field test, and data acquisition were disrupted for 2 control mice. Overall, there were no significant differences between the kindled and control mice with respect to total activity counts, total activity time, and total distances traveled, but significantly fewer static counts and shorter static time for the kindled mice than the control mice. Total rearing counts were not significantly different between the two groups, but the kindled mice had fewer central rearing counts than the control mice.

**Table 1:** Measures of the open field test.

	Control	Extended kindled	P values
Total activity counts	154.8 ± 15.9	124.5 ± 22.3	0.383
Total static counts	101.1 ± 8.9	63.1 ± 9.2	0.008*
Total mobile counts	53.7 ± 8.5	61.4 ± 16.2	0.677
Total rearing counts	26.3 ± 5.4	20.8 ± 4.5	0.444
Total central rearing counts	2.3 ± 0.7	0.3 ± 0.3	0.009*
Active time (s)	111.6 ± 11.1	92.3 ± 14.6	0.307
Static time (s)	82.8 ± 7.2	57.1 ± 6.7	0.018*
Mobile time (s)	28.8 ± 4.7	35.3 ± 9.2	0.535
Rearing time (s)	39.3 ± 7.3	27.5 ± 5.6	0.218
Inactive time (s)	188.4 ± 11.1	207.7 ± 14.6	0.307
Distance traveled (m)	10.3 ± 1.2	11.3 ± 2.5	0.791

Data (means ± SEM) were collected from 10 control mice and 9 extended kindled mice with SRS. Total counts and times were summed from a 1-h monitoring period. Significant differences between the control and extended kindled mice were denoted by *.

**Figure 4:**
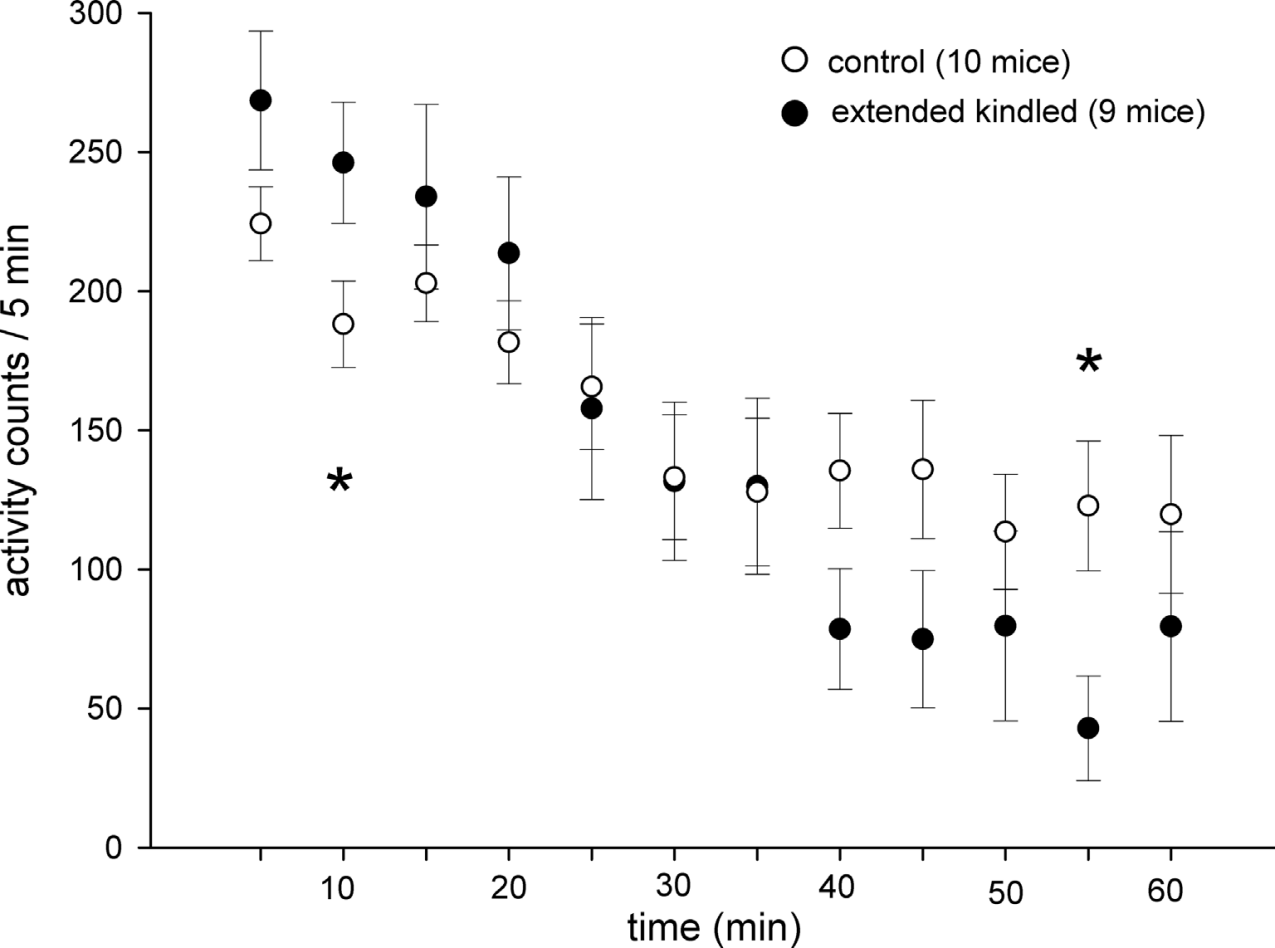
Time-dependent changes in open field activities. Data (means ± SEM) were collected from 10 control mice and 9 extended kindled mice with SRS. Individual mice were monitored for 1 h in the open field arena. The activities detected from each 5 min period were summed and presented sequentially. Significant differences between the control and extended kindled mice were denoted by *.

The kindled and control mice showed similar acclimation during the 1-h open-field test. When open-field activities were analyzed every 5 min, time-dependent decreases in activity were evident for both the kindled and control mice. Significantly higher or lower activities in the kindled mice were noted only in 5–10-min or 50–55-min periods (**Figure 4**).

## Discussion

Old age is associated with high incidence of seizures and epilepsy. Temporal lobe epilepsy is the most common type of epilepsy seen in the adult and aging populations. While stroke, dementia, and brain tumors are recognized risk factors, the etiology is unknown for many aging/aged individuals with new-onset epilepsy (Hauser, 1992; Brodie et al., 2009; Ferlazzo et al., 2016). It is therefore important for pre-clinical studies to examine epileptogenesis and comorbidities in middle-aged/aging animals (Kelly, 2010), but such studies remain limited (de Toledo-Morrell et al., 1984; Fanelli and McNamara, 1986; Hattiangady et al., 2011; Stover et al., 2017). Currently, there is no published information concerning hippocampus-dependent memory functions in epileptic middle-aged/aging animals with epileptic seizures. Here, we provide original information in this area using a mouse model of extended hippocampal kindling.

There were four main observations in our present experiments. 1) Both the kindled and control mice demonstrated decreases in time and distance to find the platform in the visible trials, suggesting that they could learn the task. 2) The kindled mice performed significantly worse than the controls in both the visible and hidden platform trials of the MWM test compared to control mice. 3) The kindled mice crossed the pervious location of the hidden platform fewer times than the control mice during both probe tests and had a memory deficit as measured by time and distance spent in the correct quadrant during the first probe test but not the second probe test. These observations suggest that, with increased training, the deficit in kindled mice may be lessened. 4) The kindled and control mice demonstrated similar locomotor activity in the open-field test as measured by total activity count and total distance traveled, but evident anxiety-like behaviors were noticeable in the kindled mice as they had fewer central rearing count and more static count and time (Seibenhener and Wooten, 2015). Overall, these observations suggest that the extended kindled mice have a modifiable deficit in hippocampal-dependent learning and memory in the MWM test.

The spatial learning and memory deficit we observed from mice following extended hippocampal kindling appear to be more pronounced than that recognized in adult rats following extended amygdala/performant-path kindling (Cammisuli et al., 1997). This may be due to multiple experimental factors including differences in animal species and ages (Löscher et al., 2017), kindling sites, SRS, and interictal spike incidences. Specifically, we conducted hippocampal kindling in middle-aged C57 black mice (11–13-month-old). C57 black mice ages 8–16 months have been shown to perform poorly relative to younger mice of 3–6 months old in the MWM test (de Fiebre et al., 2006; Shoji et al., 2016; but see Benice et al., 2006; Boyer et al., 2019). A potential memory decline in the middle-aged mice used in our experiments may partly count for the moderate improvement of the control mice as well as the non-improvement of the kindled mice during the hidden platform trials. In addition, SRS with mean incidences of 6–7 events per day were observed from the extended kindled mice before and following the MWM tests, whereas SRS incidences were variable in the amygdala/performant-path-kindled rats (Cammisuli et al., 1997; Michael et al., 1998). As pronounced deficits in spatial learning and memory have been observed in other epilepsy models with SRS (Scantlebury et al., 2005; Brandt et al., 2007; Gröticke et al., 2008; Müller et al., 2009; Seeger et al., 2011; Niquet et al., 2016; Pascente et al., 2016; Vrinda et al., 2017), it is likely that frequent SRS in the extended kindled mice may lead to more severe impairment in spatial learning and memory. Moreover, frequent interictal spikes were consistently observed from extended kindled mice before and following the behavioral tests. These interictal spikes might also have strong detrimental impacts on hippocampal cognitive process (Holmes, 2013; Gelinas et al., 2016).

Several previous studies have examined the effects of hippocampal kindling on spatial learning and memory in adult rats (Gilbert et al., 1996; Leung et al., 1996; Sutherland et al., 1997; Gilbert et al., 2000; Hannesson et al., 2001a, Hannesson et al., 2001b; Hannesson et al., 2004; Leung and Shen, 2006). Fully kindled rats performed more poorly than controls in visible and/or hidden platform trails but not in probe tests (Gilbert et al., 1996; Gilbert et al., 2000) or showed deficits in hidden platform trials but not in visual platform trials and probe tests of the MWM test (Hannesson et al., 2001b). Hippocampal kindling did not affect performance (hidden platform trails) in the early phase of a delayed-match-to-place maze test but disrupted performance during the delay phase of the test (Hannesson et al., 2001a). Partial kindling did not affect initial acquisition in the MWM test or a radial-arm maze test but induced deficits in rats that were trained prior to kindling and retested after variable delays (Leung et al., 1996; Sutherland et al., 1997; Leung and Shen, 2006). There are noticeable differences between these studies and our present observations with respect to performance in the MWM test. In particular, extended kindled mice performed poorly relative to controls in visible and hidden platform trials and in the first probe test, whereas fully kindled rats showed variable impairments in these measures. In addition, extended kindled mice did not show trial-dependent improvement in the hidden platform trails, whereas such improvement was evident in fully kindled rats (Gilbert et al., 1996; Gilbert et al., 2000; Hannesson et al., 2001b). In light of the notion that a few localized hippocampal seizures disrupted spatial cognition (Hannesson et al., 2004) and cause distributive alterations of the entorhinal–hippocampal circuit responses (Leung and Shen, 2006), cumulative seizure activities induced during extended kindling, in addition to persistent SRS and interictal spikes, may worsen spatial learning and memory outcomes in our model.

Other studies have conducted long-term kindling (66–99 stimulations) of the amygdala, hippocampus, or caudate nucleus in adult rats (Kalynchuk et al., 1998; Magyar et al., 2005; Fournier et al., 2013). While the long-term kindling did not induce SRS, kindled rats presented deficits or abnormalities in open-field activity, fear memory, and/or sexual behaviors. It remains to be tested whether similar behavioral deficits/abnormalities occur in extended kindled mice with SRS.

Our present experiments have some limitations. Regarding the MWM test, we did not analyze swim trajectories due to errors in data acquisition, which prevents assessment of whether the kindled mice employed different strategies relative to the control in searching the platform. In addition, middle-aged C57 black mice, particularly those experienced extended hippocampal kindling, may have visual impairments (Vorhees and Williams, 2006), which might complicate their performances in the visible platform trials. The uses of other spatial tasks less dependent upon visual cues than the MWM test may help address this complication in our model. Furthermore, we used a small open-field arena relative to those employed in other studies (see review by Kraeuter et al., 2019). The impacts of this confound on our present observations need to be verified. The lack of assessment of a causal relation between SRS and spatial learning and memory deficit is a major weakness of our present study, which is particularly important for exploring potential management strategies for alleviating epilepsy comorbidities. Despite these limitations and weaknesses, it is our hopes that our present works may help future studies that examine epilepsy comorbidity in middle-aged/aging animals using the mouse model of extended hippocampal kindling.

## Data Availability

All datasets generated for this study are included in the manuscript/supplementary files.

## Ethics Statement

The animal study was reviewed and approved by Animal care committee of University Health Network.

## Author Contributions

HL, KS, HS, and LZ participated in experimental design, data discussion, and interpretation. DW and JE participated in data discussion. NS, JC, CC, YL, SL, CW, KS, and HMS conducted experiments and/or data analysis. KS and LZ participated in manuscript assembling and writing.

## Funding

This work is supported by research grants from Natural Science and Engineering Research Council of Canada (RGPIN-2015-04153) and Eplink of Ontario Brain Institute.

## Conflict of Interest Statement

The authors declare that the research was conducted in the absence of any commercial or financial relationships that could be construed as a potential conflict of interest.
